# Luxembourg and Ireland in global financial networks: Analysing the changing structure of European investment funds

**DOI:** 10.1111/tran.12517

**Published:** 2022-01-19

**Authors:** Dariusz Wójcik, Michael Urban, Sabine Dörry

**Affiliations:** ^1^ School of Geography and the Environment Oxford University Oxford UK; ^2^ Department of Urban Development and Mobility Luxembourg Institute of Socio‐Economic Research (LISER) Esch‐sur‐Alzette Luxembourg

**Keywords:** financial centres, financial geography, global financial networks, investment funds, Ireland, Luxembourg

## Abstract

Using a unique database on investment funds and the conceptual framework of global financial networks, this paper examines the spatial structure of the European investment fund industry, with particular focus on Luxembourg and Ireland. Grounded in financial and economic geography, the paper shows how these countries became the leading investment fund domiciles through a mixture of structural factors and agency enabling a fast and flexible implementation of the European Directive on the Undertakings for the Collective Investment in Transferable Securities (UCITS) of 1985, and the cultivation of the investment fund industry ever since. In the process, Luxembourg and Ireland have built on and developed their functions as offshore jurisdictions and international financial centres, both sustained by their governments and regulatory agencies. The analysis of the functional structure of investment funds and their networked geography reveals the increasingly dominant position of London as the investment management centre for the industry, and the increasing concentration of control by large asset management firms. Stripped to its basics, the geography of European investment fund networks is about large, mainly US, asset management firms, creating and managing funds in Luxembourg and Ireland, and investing money through London. As such, the rise of European investment funds can be seen as an example of European financial integration through Americanisation. The Luxembourg and Irish investment fund industry are connected mainly through London and New York, and thus function as satellites of the NY–LON axis, rather than a Luxembourg–Dublin axis in international finance. Overall, the paper demonstrates that studying this seemingly arcane industry, and the role of two small countries in it, reveals much about the nature of financial globalisation.

## Graphical abstract

Using a unique database on investment funds and the conceptual framework of global financial networks, this paper examines the spatial structure of the European investment fund industry. We show the growing importance of Luxembourg and Ireland in the industry, driven by European financial integration, which in turn is led by US banks and asset management firms rather than European firms. We demonstrate that the US influence on the European investment fund industry has operated through the New York–London axis, with Luxembourg and Ireland functioning increasingly as its satellites.

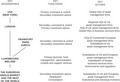

## 1 INTRODUCTION

Asset management is a large and growing industry at the centre of global finance, investing the money of individuals and institutions (such as banks, insurance companies, pension funds, family offices and Sovereign Wealth Funds) in diversified assets (mainly equity and debt securities). As such, it plays a crucial role in investment chains connecting savers with companies and governments issuing securities (Arjales et al., 2017). The key vehicle of the asset management industry is an investment fund (IF) used to pool investors money to invest in a clearly defined set of assets and following a clearly defined strategy. Through hundreds of thousands of IFs, focusing on various asset classes, sectors, geographical markets, and investor groups, the industry manages approximately US$90 trillion worth of assets globally (BCG, 2020). Demand for asset management services has risen in the last 40–50 years as a result of broader processes, including the retreat of the welfare state in advanced economies, accumulation of wealth in emerging economies, as well as individualisation of risk globally (Langely, 2008). At the same time, asset management has become an important employment and income generator for cities and countries that host the asset management industry (Clark, 2000a, 2000b).

Geographers have made major contributions to understanding asset management, its position in global finance, and impacts on economy and society. Key studies have focused on the behaviour of institutional (Clark, 2000a, 2000b; Clark et al., 2013) and individual investors (Clark & Knox‐Hayes, 2007), relationships among various groups of actors in the investment chains (Clark & Monk, 2017; Urban, 2019), the geography of command and control functions of asset management firms (Bodenman, 2000; Haberly et al., 2019), the structures of the asset management industry in international financial centres (IFCs) (Dörry, 2015), employment structure (Urban, 2018; Wójcik & Cojoianu, 2018), as well as impacts of asset management on everyday life (Langley, 2008). This literature shows how geography is present on both the production and consumption side of asset management, through IFCs and their networks, national and international regulations and institutions, as well as national, local, and corporate cultures. More work, however, is needed on how specialised asset management functions are embedded in places, resulting in distinct networks of financial activity and influence. This is important not only to better conceptualise empirical patterns of finance, but also to contribute to the political‐economic geography of finance (see Appendix S1).

To extend geographical scholarship on asset management, this paper examines the spatial structure of IFs in Europe. Based on the location of assets under management (AuM), Europe represents the second largest asset management market in the world, smaller than North America, but (still) larger than Asia (BCG, 2020). It is also more integrated and internationalised than any other regional market. While geographical analyses of asset management have focused on New York and London as IFCs, Luxembourg and Ireland are the second and third largest IF domiciles in the world after the USA. In this context, the paper is guided by two research questions:

1. What is the spatial structure of European IFs and how has it evolved over time?
2. What factors explain the position of Luxembourg and Ireland in this structure?

The paper offers contributions that go beyond understanding IFs and asset management themselves. By focusing on Luxembourg and Ireland, we add to the surprisingly small body of literature on the position of these countries in international finance (Dörry, 2016; Majerus & Zenner, 2020), as well as to rare comparative studies of financial centres (Engelen & Grote, 2009; Faulconbridge, 2004; Sassen, 2001). We show that the growing importance of Luxembourg and Ireland in the IF industry is driven by European financial integration, which in turn is led by US banks and asset management firms rather than European firms. As such, we contribute to studies on European financial integration through Americanisation (e.g., Wójcik, 2002), understood as the outsized role of the USA in global finance benefiting private US capital and the geopolitical interests of the US government (e.g., Gowan, 1999). More specifically, we demonstrate that the US influence on the European IF industry has operated through the NY–LON axis, with Luxembourg and Ireland functioning increasingly as its satellites. In summary, we prove how studying this seemingly arcane industry, and the role of two small countries in it, reveals much about the nature of European financial integration and the broader processes of financial globalisation.

The paper proceeds by outlining the functional and spatial structure of IFs before explaining the dataset behind our analysis. Empirical sections start with documenting the rise of Luxembourg and Ireland, followed by their analysis in broader networks of the IF industry. Conclusions restate our main findings and offer future research directions.

## 2 THE FUNCTIONAL AND SPATIAL STRUCTURE OF INVESTMENT FUNDS

We analyse European IFs through the lens of global financial networks (GFNs), which can be defined as networks of financial and business services (FABS; including accounting and law), governments, IFCs, and offshore jurisdictions (OJs) closely interlinked with each other at the heart of the world economy (Wójcik, 2018; Wójcik & Camilleri, 2015). This framework helps us consider the industry as not just the realm of financial firms, but also other advanced business services, including accountancy and law firms (Dörry, 2015). Second, we include the role of governments and inter‐governmental organisations in the analysis, as actors setting the rules that shape the opportunities and constraints for how the industry operates, also geographically. Third, we examine how asset management activity manifests itself in IFCs and OJs, the former concentrating the command, control, and investment functions, while the latter attract registrations of financial vehicles. To be sure, there is expertise and services involved in both IFCs and OJs, and so the distinction between IFCs and OJs is not binary with the same places sometimes serving both functions (Dörry, 2015; Hudson, 2000; Wójcik, 2013). Finally, we consider how the above key actors (FABS and governments) and territories (IFCs and OJs) are interlinked with each other in the context of the global economy. In a nutshell, the GFN in place since at least the mid‐20th century can be characterised by the hegemony of the US political and financial power projected through US‐dominated FABS and the NY–LON axis of IFCs, extended through a network of OJs, themselves mostly former British colonies (Wójcik, 2013, 2015).

To set the stage for the examination of European IFs as a subset or a subnetwork of GFNs, we delve deeper into their functional structure, building on Clark's pioneering work mapping the functions of pension fund investment management (2000b). Understanding the functional structure helps understand the spatial structure of any industry, but this is particularly true for the financial sector, where the intangible nature of products and services adds to the complexity, with formal differences obscuring functional similarities (Dixon, 2012).

An IF is an entity that pools investors money to provide them with professional investment management. A fund sells its shares (also called units) to investors and invests the proceeds, mostly in securities, to achieve its stated investment objectives. Net asset value (NAV) per share is calculated at least daily, after deducting all costs and fees incurred by the IF. It determines the price at which shares can be redeemed by existing investors and sold to new investors.

**FIGURE 1:**
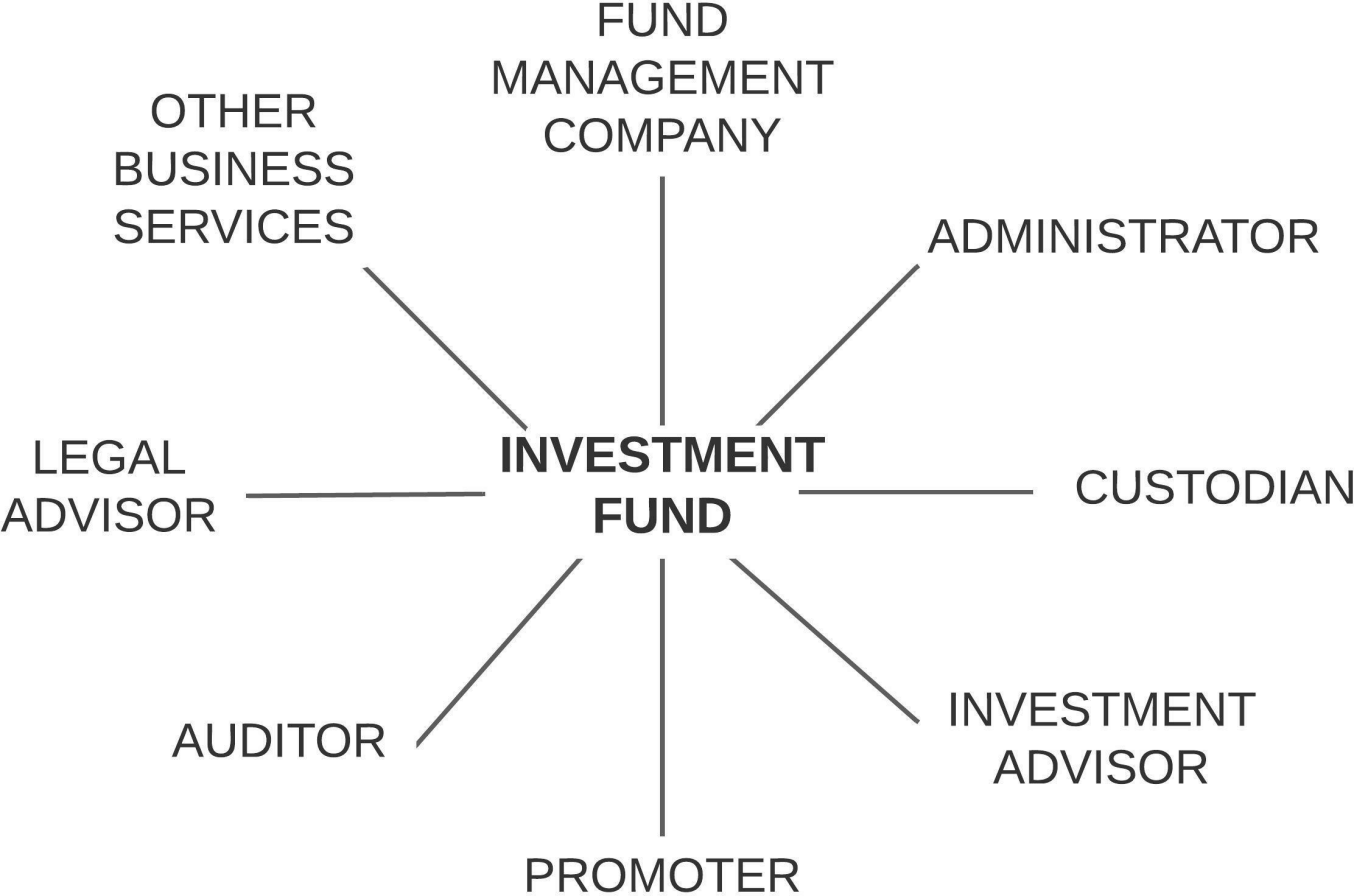
Functional structure of an investment fund *Source*: Authors

As Figure 1 illustrates, multiple entities are involved in the operations of an IF. A legal entity called a fund management company (FMC) creates an IF, applies for its registration, and is responsible for managing legal and financial aspects of the fund, including the appointment of companies servicing the fund. An IF has no parent company, and an FMC manages rather than owns an IF. Assets of an IF are entrusted to a custodian for safe‐keeping and ensuring that transactions in units and the calculation of NAV take place in accordance with the IF rules and appropriate laws. An administrator maintains the unit‐holder register, handles unit issues, redemptions, and customer inquiries, as well as accounting and compliance monitoring. An investment advisor is responsible for investing assets in the fund portfolio. The fund is marketed to potential investors by a promoter. Every fund needs to have a legal advisor and be audited to verify if the financial reports prepared by the administrator are a true and fair representation of the funds actual state. Finally, IFs are supported by a wide array of other FABS services, from tax, accounting, IT, strategy, HR, real estate, and travel consulting, to building and personnel security. Figure 1 shows a stylised IF structure, as actual structures differ depending on jurisdiction and IF type (see e.g., Dörry, 2015; PwC, 2020).

There are significant economies of scale and scope involved in IF activity. Given the uncertainty of investment returns and difficulty (if not impossibility) of outperforming average market returns (Clark, 2000a, 2000b), cost minimisation is crucial. Many costs involved in IF setup and management, including IT and regulatory compliance, are largely fixed, with larger assets under management lowering average costs. Economies of scale apply particularly strongly to custody and administration, as the most high‐volume, low‐value activities. As a result of economies of scale and scope, the IF industry has become dominated by large asset management companies that combine various functions through specialised subsidiaries (Haberly et al., 2019). Regulations can prevent certain combinations of functions. Most commonly, custody of assets is not allowed to be done by an FMC. Major economies of scale and scope are also present in advisory functions, with the big four audit and consulting companies (Deloitte, EY, KPMG, PwC) offering a whole range of services to IFs, from accounting and audit, through tax, IT consulting, and HR.

As a legal entity, an IF has to be legally incorporated (domiciled) in a jurisdiction, which determines the laws and regulations it is subject to. In practice, it is an FMC that applies for the registration of an IF in a jurisdiction. In the EU, IFs are subject to the UCITS Directive, first adopted in 1985, as the main framework covering collective investment schemes for small investors and the Alternative Investment Fund Management Directive for non‐UCITS IFs, including hedge funds and private equity, first adopted in 2011. The key advantage of both directives is that an IF authorised in one Member State can be sold to investors in any Member State. Put differently, the directives offer IFs a single European passport, offering EU‐ and non‐EU‐based firms unprecedented access to EU markets. In addition to the EU‐wide directives and regulations of the European Securities and Markets Authority (ESMA), IFs in the EU are subject to national laws and regulations of the countries in which they are domiciled. These can vary in their interpretation of the directives and EU‐wide regulations, ranging from liberal implementations to gold plating, which involves additional regulation, typically raising the costs for business.

The functional structure of IFs guides our analysis of their spatial structure. We start with the domicile of IFs. This takes us to the realm of OJs, as we would expect IFs to be registered in jurisdictions that offer legal, regulatory, and tax advantages. However, this does not rule out the possibility that the same jurisdictions can provide other IFC functions for IFs. The EU legal framework relies on the regulation of IFs by the country of their domicile, with certain functions required to be performed in that country. This co‐location determined by law is enhanced by economies of agglomeration, whereby some functions are located in the same country because other functions are already there. In this context, our task is to examine what OJ and IFC characteristics helped the rise of Luxembourg and Ireland as leading IF domiciles, and what has been the role of governments and FABS in the process. After examining domicile, we will investigate the changing networked geographies of key IF functions by country, the firm‐level concentration across these functions, as well as the origin and structure of leading firms in the industry.

## 3 DATA

We sourced data from a proprietary database of Refinitiv Lipper for Investment Management, a world‐leading provider of information and analytics on IFs. The database covers all types of IFs, from mutual funds and closed‐end funds, through exchange‐traded funds, investment trusts, and IFs set up by pension funds and insurance firms to hedge funds. Geographically, our sample covers IFs domiciled in any European Single Market (ESM) country (i.e., one of the 28 EU member countries, plus Switzerland, Liechtenstein, Norway, and Iceland). After cross‐examining the dataset against alternative sources, we established that the coverage of hedge funds is not reliable enough, and hence we excluded that category from our quantitative analysis. We also concluded that comprehensive data coverage starts in 2003, which led us to focus on the period between year‐ends 2003 and 2019. In total, this gives us a sample of 294,056 IFs active at least at some point in this period.

In addition to data on the domicile of IFs and AuM, Refinitiv Lipper offers information on the currency in which each IF and its segment are denominated, types of assets they invest in, and the geographical focus of investment. Crucially, it also contains information on the name of the FMC, administrator, custodian, investment advisor, and promoter of each IF, as well as phone numbers for some of them. Where phone numbers were available, we matched the country code with the country name. For thousands of companies (approximately a third of all IF service providers) for which phone numbers were missing, we manually collected the country of operational headquarters using a variety of sources, including Orbis, Bloomberg, Nexis UK, and corporate websites. Given how time‐consuming such manual data collection is, we only conducted it for years 2003 and 2019. Since phone numbers for promoters were missing for an absolute majority of firms, we could only collect data on the nationality (country of operational headquarters) of the largest firms. In some cases, the nationality of administrators, custodians, FMCs, and investment advisors could not be established through a manual search. These firms, however, represented less than 1% of AuM in 2003 or 2019, and hence the impact of missing data thereon is negligible.

## 4 THE RISE OF LUXEMBOURG AND IRELAND AS IF DOMICILES

**FIGURE 2:**
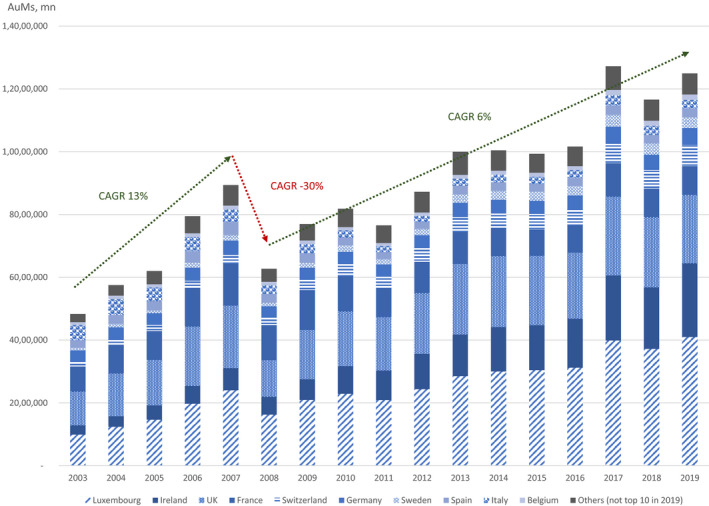
Evolution of fund domicile in the European Single Market, 2003–19 *Source*: Authors based on data from Refinitiv Lipper for Investment Management. CAGR, Compound annual growth rate

As Figure 2 shows, between 2003 and 2019, AuM of IFs domiciled in the ESM have grown from $4.8tn to $12.5tn. In 2003, the UK was the leading domicile, followed by Luxembourg, France, Italy, Germany, and Ireland. In relation to GDP, Ireland and particularly Luxembourg had thus established themselves as specialised centres of IF domicile by the early 2000s. By 2019, Luxembourg and Ireland doubled their market shares by over 12 percentage points each, doubling their joint market share to over 50%. France, Italy, the UK, Germany, and Spain lost between 3 and 9 percentage points of market share. In this section, we explain the roots of this change in the process of European financial integration, starting with and focusing on the pre‐2003 history, and finishing with developments since 2003, which will be explained further in the following section.

Key to the early growth of both Luxembourg and Ireland as IF domiciles was a quick implementation of the 1985 UCITS Directive. Prior to 1985, cross‐border asset management in Europe was a relatively small industry, focusing mainly on private wealth management, with London, Switzerland, and Luxembourg as its key centres. The management of assets of retail investors, however, was a domestic affair, with IFs and associated functions located in the same country as the country of their investors (Story & Walter, 1997). The 1985 UCITS Directive was path‐breaking in this respect and a milestone of European financial integration. By facilitating collective investments in securities, it stimulated the growth of IFs and laid foundations for an integrated market for the production and distribution of IF services. The Directive meant that new IFs could be registered in, and existing IFs relocated to, Member States that were not the main markets for the distribution of these funds. Luxembourg was the first country to implement the Directive in 1988, Ireland came second in 1989. Both countries implemented it in a flexible manner, with no gold plating, and with the purpose of becoming centres of the IF industry. At that point, however, there was a major difference between them. While Luxembourg was already a major OJ and an IFC, Ireland had little experience of the former and none of the latter.

Luxembourg's pioneering role in European economic integration has deep roots, including an economic union and common currency with Belgium dating back to the 1920s. The country has a long history of attracting international business through regulation and taxation, including the 1929 Holding Company Regime, and plentiful treaties to avoid double taxation, facilitated by its OECD membership (Majerus & Zenner, 2020). In the post‐Second World War decades, Luxembourg established itself as a centre of private wealth management (led by KBL bank), Eurobond issuance and listing, as well as infrastructure for clearing and settlement of cross‐border financial transactions. Small size, political and economic stability, central location in Europe, and a multilingual workforce were all major factors behind these developments, as was the proximity between financial elites and the government. In the 1970s and 1980s, the key role here was played by Philippe Duvieusart and his collaboration with Pierre Werner. Duvieusart was son of the former Belgian Prime Minister and had a US PhD and work experience in the World Bank Group. He ran KBL and was instrumental in the development of the Eurobond market. Werner, a banker himself, later became the Finance Minister and Prime Minister of Luxembourg (Dörry, 2016). In the mid‐1980s, Luxembourg was thus ready to seize the opportunity created by the UCTIS Directive, while a slowdown in Eurobond market activity caused by the developing country debt crisis only increased capacity and motivation for capitalising on it.

While Ireland joined the European Economic Community (EEC) in 1973, it had a long history of financial integration with (or colonisation by) the UK (OConnor, 2020), including a common currency and a stock exchange. By 1985, it had become a major FDI destination, building on its advantages of the English language, common law, Irish diaspora in the USA (where most FDI came from), location between the USA and the UK, a long list of double tax treaties (though not as long as Luxembourg's), and increasing access to European markets enabled by the EEC. As such, Ireland already had some features of an OJ, exemplified by tax breaks offered to foreign firms in the Shannon Airport Area (White, 2005). However, despite the interest of foreign banks coming to Ireland to serve foreign firms, a large Irish community of professionals in the City of London, and efforts of the world‐famous inward investment promotion agency IDA, in the mid‐1980s Dublin was anything but an IFC (Murphy, 1998). Seeing an opportunity to change this situation, Dermot Desmond, a businessman with a career spanning Citibank, PwC, brokerage, and private equity, and Michael Walsh, with a US PhD and experience at Wharton Business School, then a professor of finance and banking at University College Dublin, introduced the idea of developing a centre for international financial services in Ireland to Charles Haughey, the leader of the opposition party Fianna Fáil, who included it in his manifesto for the general election of 1987. After winning the election and becoming the head of government (Taoiseah), Haughey set up the International Financial Services Centre Committee involving government departments and the private sector, and designated the Dublin Docklands as the site for the International Financial Services Centre (IFSC).^1^

The big attraction of the IFSC located in Dublin's Docklands was a 10% corporate income tax (CIT) rate, a fraction of CIT rates around Europe and the 40% rate applying in the rest of Ireland at that time. IFs became one of the sectoral foci of the IFSC, alongside cross‐border banking and insurance. Luxembourg became the main template in terms of regulation and taxation of IFs, with much learning facilitated by FABS firms, including PwC as the leading auditor and consulting services provider to IFs. It was clear that to compete with Luxembourg, Ireland had to promote itself more aggressively. A myriad of flows and stocks in IF activity can be taxable: subscriptions, redemptions, NAV, securities transactions, IF support services, and IFs themselves. CIT, personal, value‐added, inheritance, and specific taxes to financial transactions can all apply. With money coming from and going to investors in multiple countries, and assets bought and sold in multiple countries, the regulatory and tax costs and complexities are compounded. In addition to a low CIT, Ireland offered IFs all possible tax exemptions. This was exceptional, as even Luxembourg applied some taxes, including a 0.06% tax on fund assets (Oxera, 2005). Since Ireland implemented UCITS, Luxembourg also enhanced its tax incentives for IFs, e.g., reducing CIT from 34% to 30% and abolishing the trade capital tax.

While enabled by the introduction of UCITs, the rise of the IF industry in Luxembourg, and particularly in Ireland (starting from scratch), did not happen overnight. International financial markets recovered from the 1987 stock market crash only in the early 1990s. Both countries moved to adjust laws to enlarge the range of IF structures available, with a new Companies Law and Unit Trust Act in Ireland introduced in 1990. IFSC had to be physically developed, and construction funded. In 1989, IFSC was still a plan contested by the opposition to Fianna Fáil in the city government and the national parliament. Further political process was needed to include the support for financial services firms as one of the statutory goals of the Dublin Docklands Development Authority, the main entity governing the IFSC, in 1997. It took time to attract domestic and particularly foreign investors, with much effort focused on the Irish diaspora in the USA. It was only in 1992 that Chase Manhattan (part of today's JP Morgan) became the first foreign company to rent a building in the IFSC, followed by Citibank in 1993.

Several factors can explain the fast growth of IFs since the early 2000s. Creation of the Eurozone accelerated the integration of the IF industry and made the Eurozone a more attractive place for investments, while widespread securitisation provided new financial instruments to be packaged into IFs. Further regulation of IFs was equally, if not more, important. UCITS III Directive of 2001 allowed IFs to invest in a much wider range of financial instruments, including derivatives. Crucially, UCITS IV, implemented in 2011 introduced a management company passport, lifting the requirement for FMCs to be domiciled in the same country where the IF is domiciled, accelerating the process of integration. Luxembourg and Ireland were well positioned to take advantage of these tailwinds.

Though its share fell significantly, the UK remains a major IF domicile, revealing a degree of path dependence in the IF industry, related to both supply and demand factors. The UK outcompetes Luxembourg and Ireland in terms of qualified labour, quality of financial infrastructure, not to mention the liquidity of its capital market. The UK hosts Europe's biggest institutional investors, including pension funds, which generate demand for IF products (Clark, 2000a, 2000b). These factors represent forces that can keep much investment advice and promotion (distribution) of IFs in the UK, creating incentives for keeping other functions, including domicile, in the UK. In addition, UK FMCs looking for cost savings related to back and middle‐office functions have had the rest of the UK as an alternative to moving such functions abroad (Oxera, 2005). Thus, we need to see the rise of Luxembourg and Ireland as IF domicile and industry as happening against the odds of powerful factors that keep this industry within national borders of other countries, with the UK in the lead.

While separating IFs from other financial services in terms of employment is difficult, in Luxembourg at the end of 2019 there were approximately 8,000 people employed directly in investment firms and investment fund management, in addition to over 15,000 other financial sector professionals outside of banking, many of whom work in the IF industry (CSSF, 2020). In Ireland, there were over 14,000 professionals in the IF industry and over 40,000 in IFSC by the end of 2017 (IFS, 2018, 2008). PwC itself employs nearly 3,000 people in Luxembourg and over 2,000 in Dublin (PwC, 2020). Both countries have prospered as IF industry centres despite growing congestion, labour, and real estate costs. In Ireland, this has been managed partly by IFs locating their activities outside of IFSC and Dublin, particularly since the early 2000s when, under pressure from the EU, CIT rates between IFSC and the rest of the country converged to 12.5% (Hendrikse, 2013; Sokol, 2007). Luxembourg has relied increasingly on commuters from Belgium, France, and Germany for its workforce (Walther et al., 2011). Both countries have invested in financial education to upgrade skills and promoted innovation. Luxembourg has led the development of IF focusing on Sharia‐compliant products, microfinance, and sustainable finance (Dörry & Schulz, 2018), and Chinese markets, building on Luxembourg's emergence as the centre of China Dim Sum bond issuance, RMB internationalisation, the Green Bond Connect Luxembourg‐Shanghai, as well as FinTech and regulatory technology (RegTech) (CCAF & EY, 2019). The Irish IF industry has focused on securitisation and index tracking funds, including exchange‐traded funds, in which it has a 62% market share in the ESM, building on synergies with the growing FinTech sector, hedge fund industry, and aviation finance, in which Ireland has become the world's leading centre (IFS, 2017).

The 2008 financial crisis had a muted impact on the growth of the IF industry. Luxembourg saw bank bailouts arranged in coordination with Belgian authorities, but there was little direct impact on revenues in insurance, private banking, and IFs (Walther et al., 2011). In Ireland, after a property boom fuelled by Irish banks (Byrne, 2016), the crisis led to a major but short‐lived recession. A temporary fall in labour and real estate costs, if anything, offered a relief to the IF sector. In the wake of the crisis, the role of the European Central Bank and EU institutions in relation to national authorities was enhanced, but the brunt of new regulation focused on banks rather than asset management (Wójcik & Cojoianu, 2018).

In summary, Luxembourg and Ireland would not have become the dominant IF domiciles in Europe without the opportunity created by the UCITS Directive of 1985. They capitalised on their features as small, open, and well‐connected economies, ready to offer tax and regulatory advantages (as OJs) as well as IF‐specific workforce and expertise (as IFCs). While Luxembourg was a veteran in both areas before 1985, Ireland had to build its capabilities, learning from the example of Luxembourg. The role of supranational and national governments in the story thus far should be clear. While these elements of the GFN framework seem to fall into place, we can turn to examine the FABS involved, the networks of IF industry they create, and their role in European and global finance.

## 5 MAPPING THE NETWORKS OF THE EUROPEAN IF INDUSTRY

**FIGURE 3:**
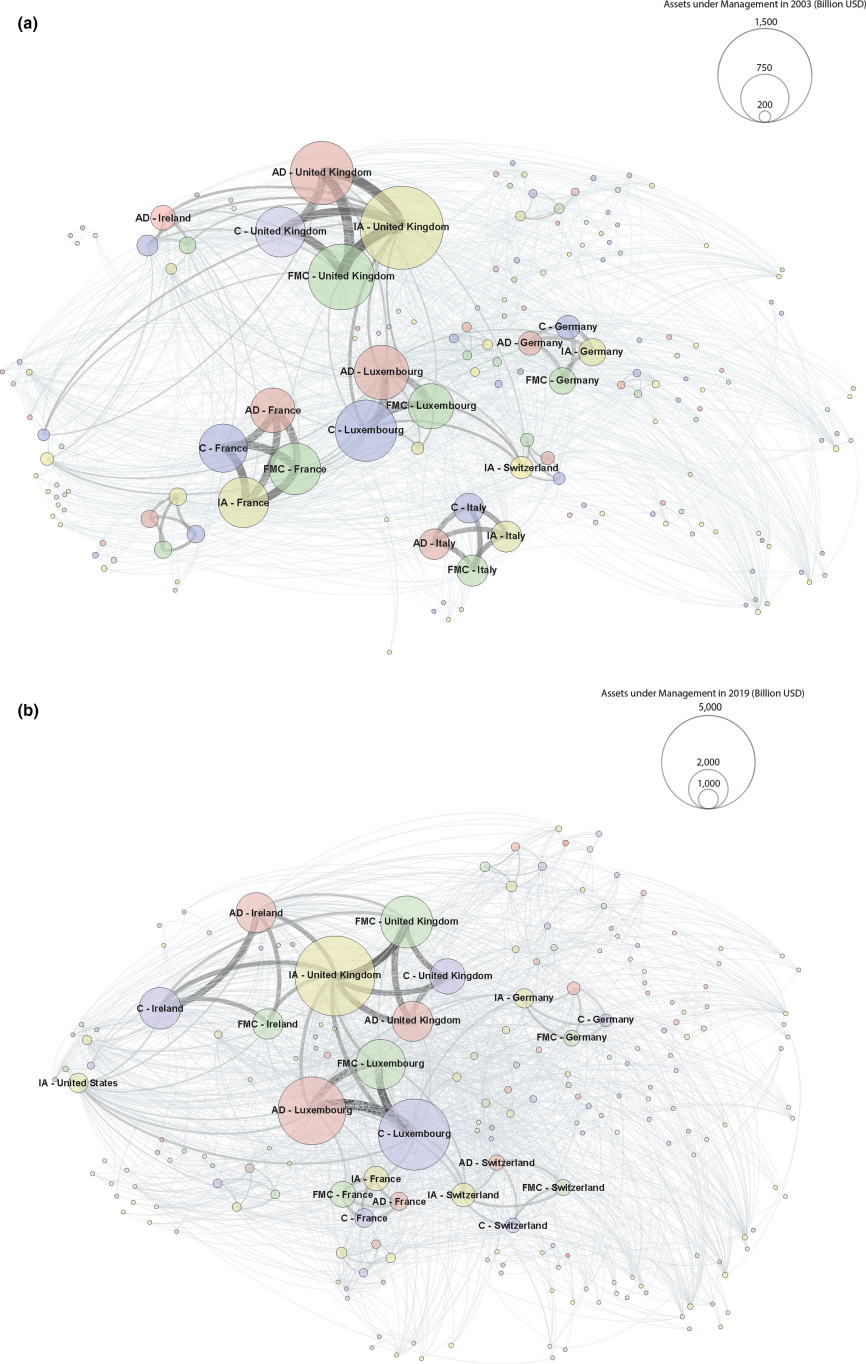
(a and b) European investment fund industry network in 2003 and 2019. *Note*: Nodes represent groups of FMCs, administrators (AD), custodians (C), and investment advisors (IA) located in a given country. A link between different nodes means that these companies serve the same IFs. The size of a node and the thickness of a link are proportionate to AuM *Source*: Authors based on data from Refinitiv Lipper for Investment Management

Figure 3 compares the network of the European IF industry between 2003 and 2019 year‐ends. Nodes represent groups of FMCs, administrators, custodians, and investment advisors located in a given country. A link between different nodes means that these companies serve the same IFs. The size of a node and the thickness of a link are proportionate to their AuM. In 2003, the UK was the dominant centre of investment advisory functions, the largest centre of administration and FMCs, and the second largest centre of custody, after Luxembourg. In contrast, Ireland appears on the network periphery, with specialisation in administration. While domestic connections dominate (particularly in France, Germany, and Italy, but also in the UK), there is a dense cloud of international connections, with Luxembourg and Ireland standing out as the most open markets.

By 2019, the industry became much more internationalised and integrated. The cloud of connections grew denser, and specialisation increased. The UK dominates investment advisory to a much higher degree than in 2003. Luxembourg absolutely dominates custody, followed by Ireland and the UK, and together with the UK and Ireland leads the way in FMC and administration. The French and German IF industries have shrunk, and the Italian subnetwork has almost disappeared. The USA emerges as a significant centre of investment advisory. Links between Luxembourg and Ireland are relatively unimportant in the big picture of international connections. Between them, Luxembourg and Ireland account for well over 80% of administration and custody of IFs domiciled in the ESM.

**FIGURE 4:**
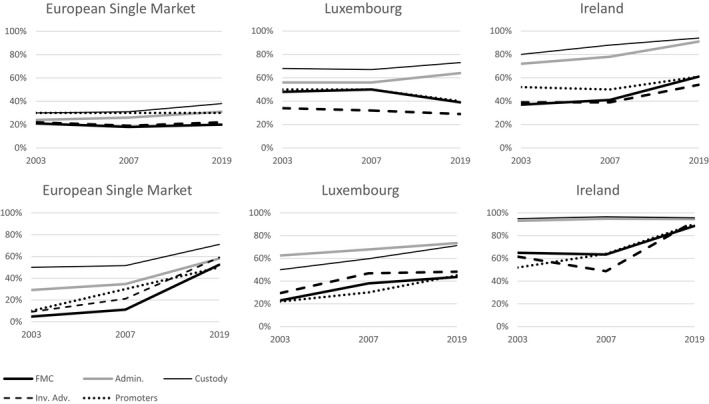
The share of the top 10 firms (top) and the share of US firms among the top 10 firms in each market (bottom) *Note*: The European Single Market includes Luxembourg and Ireland *Source*: Authors based on data from Refinitiv Lipper for Investment Management

To reveal more about the corporate structure of the IF industry and its geographical providence, Figure 4 shows the market share of the top‐10 firms and the share of US firms among the top‐10 firms. Here we should note that firms in Figure 3, grouped on the basis of location, are often (and for 2019 typically) subsidiaries of asset management firms operating in multiple countries and performing various IF services. Across the ESM, FMC and investment advisory are least, and custody (with the largest economies of scale) most, institutionally concentrated, with administration and promoters in between. In administration and custody, institutional concentration has grown over time, while in other areas it remained stable. Institutional concentration was also much higher in Ireland than in Luxembourg, due partly to the smaller size of the industry in the former. There was no company from outside North America and Europe in the top 10 in any category in the ESM as a whole, Luxembourg, or Ireland. All the top‐10 providers in any category in Luxembourg and Ireland were subsidiaries of foreign asset management firms.

The rising dominance of US firms is striking. By 2019, they were responsible for the majority of the market share of the top‐10 providers in each area of activity, from 50% in promotion to 71% in custody. In 2003, the asset management arms of banks Credit Agricole, BNP Paribas, and UBS, the UK‐based insurer Aviva, and the specialised French asset management firm Amundi, were ahead of US firms as leading IF firms in Europe. By 2019, US firms were leading in each category: Blackrock in FMC and promotion, JP Morgan in administration, and State Street in custody. This finding is line with Haberly et al. (2019), showing that while in 2006 four out of five largest asset management firms globally were European banks or insurance companies, by 2015 four out of five were US firms. European champion banks in particular were too big to be recapitalised by their governments in the wake of the global financial and Eurozone crises and were forced to sell their asset management arms (Haberly & Wójcik, 2021). The Eurozone crisis also weakened role of the Euro and the Eurozone as the focus of IF investments, with the share of the Euro falling from 60% of AuM to 45%, and the share of the Eurozone from 30% to 12% between 2003 and 2019, further disadvantaging the European asset management firms. The rise of US firms in European IF industry was also accelerated by the lifting of the co‐location requirements for FMCs and IFs by UCITS IV in 2011, and helped by economies of scale. It was fastest in custody and administration, activities with the strongest economies of scale, where US firms could apply technologies developed in their home market, being by far the largest asset management market in the world (BCG, 2020).

Luxembourg and Ireland played a key role in the Americanisation of the European IF industry. The Luxembourg industry started with a geographically diverse set of providers, with many firms coming from France, Germany, UK, Switzerland, Belgium, the Netherlands, and Italy, in addition to the USA. Between 2003 and 2019, the US firms doubled their share in top 10 in FMC and promotion and increased it significantly in other functions. In the early 2000s, for example, Deutsche Bank, with major presence in Luxembourg, sold its custody business to State Street. In Ireland, US firms dominated already in 2003, but still managed to increase their shares, reaching close to or over 90% in each category. JP Morgan, the world's largest financial company outside of China in terms of market capitalisation, and an important player in the Eurobond market, led FMC and promotion in Luxembourg. Blackrock, the world's largest asset management company, led FMC and promotion in Ireland. In administration and custody, State Street led alongside JP Morgan in Luxembourg, and dominated in Ireland.

**FIGURE 5:**
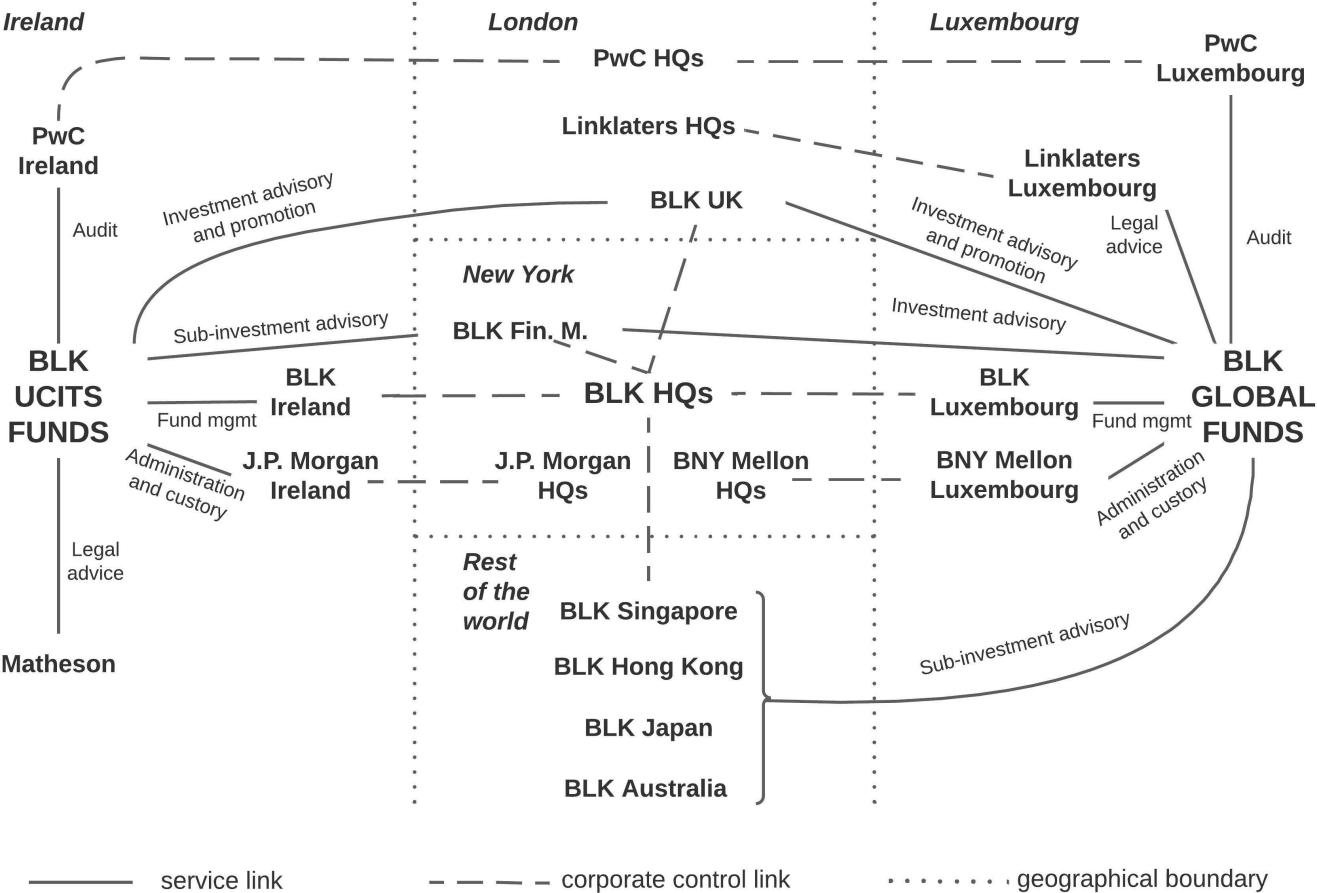
Functional and spatial structure of Blackrock UCITS and Global Funds *Note*: The structures and names of Blackrock subsidiaries and service providers have been simplified for the sake of the illustration *Source*: Authors based on data from Blackrock (2017 and 2018)

To illustrate the functional and spatial structure of the European IF industry with a specific example, Figure 5 focuses on Blackrock UCITS and Global Funds as families of IFs domiciled in Dublin and Luxembourg respectively, set up by Blackrock subsidiaries in each country. Investment advisory and promotion for both fund families is performed mainly by Blackrock's subsidiary in London but both also use its New York headquarters for investment advice; administration and custody are conducted by Irish and Luxembourg subsidiaries of US New York‐headquartered banks; audit by Irish and Luxembourg subsidiaries of London‐headquartered PwC. As Blackrock, Global Funds are invested on a more global basis than UCITS funds; they also use Blackrock subsidiaries in Singapore, Hong Kong, Japan, and Australia as investment advisors. This is also reflected in the need for more global legal expertise, sourced from the Luxembourg subsidiary of London‐headquartered Linklaters, while UCITS funds rely on legal advice from Dublin‐headquartered Matheson.

**FIGURE 6:**
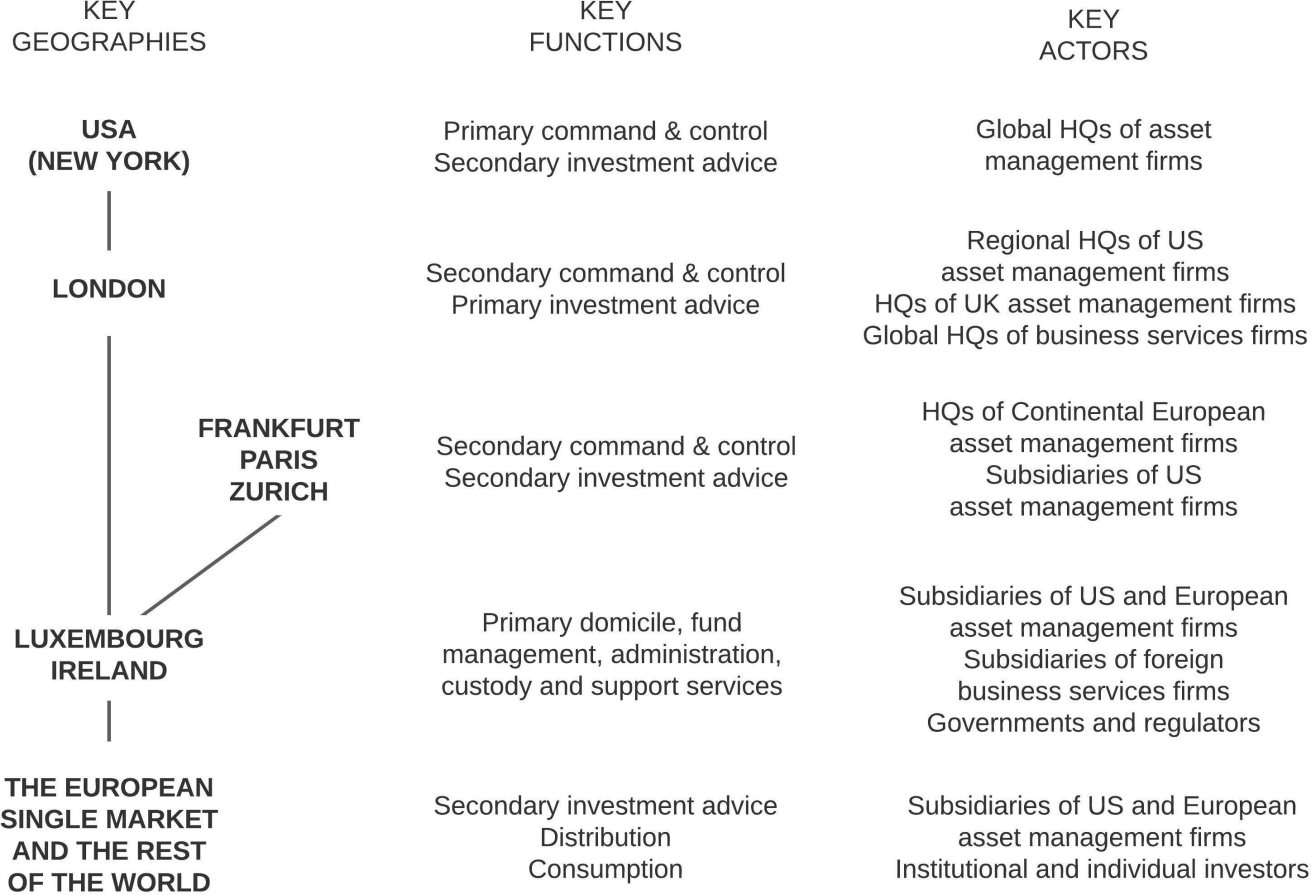
Stylised structure of the European investment fund industry *Source*: Authors

Figure 5 thus lays the ground for presenting a stylised structure of the European IF industry in Figure 6. The US asset management firms (often arms of banks), with New York as the main headquarter location, are the leading firms in the European IF industry. They establish IFs, mainly in Luxembourg and Ireland, and manage them via their subsidiaries located in those countries and in London. New York also serves as an investment advisory centre for these IFs, particularly when funds are invested beyond Europe.

London serves as a secondary command and control centre of the European IF industry, hosting regional HQs of US firms, HQs of UK firms, and subsidiaries of Continental European asset management firms, in addition to global HQs of leading business services firms serving IFs, such as PwC and Linklaters. At the same time, as Figure 3 showed, London is the primary investment management centre for European IFs, concentrating well over 90% of this activity.

Continental European IFCs, with Paris, Zurich, and Frankfurt in the lead (see Figure 3), play a secondary part in both command and control, as well as investment management. These functions are performed by HQs of Continental European firms, and subsidiaries of US and UK asset management firms located in these cities. In investment management, they complement London in areas where local knowledge and access is particularly important.

Luxembourg and Ireland (with Dublin as the dominant centre) serve as primary IF domiciles (Figure 2), and primary centres for fund management, administration, custody, and support services (Figure 3). They perform these functions through subsidiaries of US and European asset management and business services firms operating in close collaboration with the national governments and regulators. Given the role of New York and London as the capitals of the global and European IF industry respectively, Luxembourg and Ireland function as satellites of the NY–LON axis of global finance.

Finally, the ESM as a whole and the rest of the world serve primarily as markets for the consumption of IF products by investors, reached through distribution channels consisting of subsidiaries of US and European asset management firms scattered around the world. According to Deloitte (2018), Luxembourg and Ireland accounted for nearly 90% of European IFs set up for cross‐border distribution, with Singapore and Hong Kong as the main non‐ESM destinations, followed by Chile, Taiwan, Korea, and Macau. Sometimes, particularly when funds are invested globally (Figure 5), the IFCs beyond Europe and the USA can also act as secondary investment management centres.

## 6 CONCLUSIONS AND IMPLICATIONS

We have used a unique database and the GFN conceptual framework to examine the spatiotemporal structure of European IFs, with particular focus on Luxembourg and Ireland. Both countries became leading IF domiciles through a mixture of structural factors and agency enabling a fast and flexible implementation of the UCITS Directive of 1985, and the cultivation of the IF industry ever since. Luxembourg had a head start as an established OJ and an IFC, but Ireland developed capabilities in both areas by learning (mainly from Luxembourg) and promoting itself more aggressively. Importantly, European and domestic legislation and regulation did not allow excessive separation between IFCs and OJs serving IFs, requiring that domicile be accompanied with the execution of (at least) some fund management, administrative, and custodial functions, while investment advisory could be performed elsewhere. This legal geography of European IFs, with rules affecting location and co‐location, was crucial in shaping its economic geography, with Luxembourg and Ireland establishing themselves as leading domiciles and IF industry centres, with London as the absolutely dominant centre of investment advisory. In the process, the role of European countries other than Luxembourg and Ireland as IF domiciles, and cities other than London as centres of investment advisory, has declined. One lesson here is that studying the economic geography of an industry requires a thorough understanding of its functional structure as well as relevant laws and regulations, however “technical” those may be.

The story of European IFs is part of the process of European financial integration. A set of domestic markets, each with its own domicile, investment advisors, and other service providers, was being replaced with an integrated market (ESM), with Luxembourg, Ireland, and London as its specialist hubs. Increasing amounts of money from all over Europe, but also from beyond, are assembled in vehicles registered, accounted for, managed, and regulated in Luxembourg and Ireland, and invested globally by investment firms in London. The investigation of FABS involved – their origin, structure, and networks – helps reveal a progressive Americanisation of the European IF industry, with large US asset management firms creating and managing IFs in Luxembourg and Ireland, and investing money through London. As such, in political‐economic terms, it is another instance of European financial integration through Americanisation (Wójcik et al., 2018). The process has roots that go beyond the acceleration of European financial integration of the 1980s. Just as first Eurobonds were issued and booked in Luxembourg but traded in London (Weeks, 2020), IFs are domiciled in Luxembourg and Ireland with their assets traded in London.

Having two leading IF domicile and services centres creates an environment in which Luxembourg and Ireland compete with each other to offer FABS the best combination of OJs and IFC features. Luxembourg and Ireland complement each other as IF centres from the perspective of FABS firms, but not in the sense of one centre needing the other in order to function. While both countries have developed some specialisation in different of types of IFs and markets, each can assemble IFs from start to finish. Most US and other leading firms creating and serving IFs are active in both centres, but they do not really need both centres to manage any given IF. This illustrates a more general point regarding the nature of relationships between IFCs. The presence of the same FABS firms in two different cities does not automatically imply any flows, transactions, or collaboration between these cities (Pažitka et al., 2021). To be sure, we are not arguing that there are no such connections between Luxembourg and Ireland. As we mentioned, consulting firms, including PwC, played a role in sharing Luxembourg's OJ and IFC experience with Irish authorities. No doubt, there are many IF professionals moving between Dublin and Luxembourg for jobs. While the nature of magnitude of these relationships would require further investigation, what we argue here is that the Luxembourg and Irish IF industry are connected mainly through London and New York, functioning as satellites of the NY–LON axis, rather than forming a Luxembourg–Dublin axis in international finance.

There are several directions in which research in this paper could be extended. The GFN approach applied herein leaves room open for more thorough historical accounts, and more detailed accounts of the role of tax and regulation in shaping the landscape of European IFs. The political‐economic dimension also warrants further investigation, which could focus more on the role of the US political and financial hegemony and the regulatory capture of the Irish and Luxembourg state apparatus by the financial sector (with US firms in the lead). Future studies could also examine the exports of IF products to Asia, and the application of the European legislative and regulatory models for IFs in Asia, particularly ASEAN, and its consequences for financial geography of the region. What will be ASEANs equivalents, if any, of London, Luxembourg, or Ireland?

The geography of European IFs is also being reshaped by Brexit. The overwhelming concentration of investment management in London at the end of 2019 suggests that Brexit has not yet undermined London's role in the European IF industry. With the UK leaving the EU in 2020 with no comprehensive agreement for financial services trade, it is highly uncertain whether and on what conditions the European regulators, with ESMA in the lead, will allow London to be the hub of the most profitable parts of the IF industry in the ESM, of which the UK is no longer a part. Evidence is emerging that some asset management firms are moving some assets and small numbers of employees to the EU, with Luxembourg and Dublin in the lead (EY, 2021). The significance of the NY–LON axis to Luxembourg and Ireland, documented in this paper, suggest that such trickles are unlikely to turn into a flood, although it also hints at the possibility of their more direct reliance on New York, with a lesser role for London in the future. One way or another, there are many reasons for geographers to stay tuned to the developments in this seemingly arcane industry, and continue uncovering its structures and position in global finance.

## Supporting information


Appendix S1

## Data Availability

The data that support the findings of this study are available from the corresponding author on reasonable request.

## ENDNOTE

^1^
A detailed description of the origin of the IFSC can be found on the website of Finance Dublin at http://www.financedublin.com/supplements/15th_anniversary/ (accessed November 2021).
