# Supplementary material for: Luxembourg and Ireland in global financial networks: Analysing the changing structure of European investment funds
Source: Trans Inst Br Geogr. 2022 Jan 19;47(2):514–28. doi: 10.1111/tran.12517 (PMC9305508; doi:10.1111/tran.12517)
Supplement: Supplementary file 1 — Appendix S1 [file TRAN-47-514-s001.docx]

Table 1. List of acronyms

AuM assets under management

CIT corporate income tax

EEC European Economic Community

ESM European Single Market

ESMA European Securities and Markets Authority

FABS financial and business services

FMC fund management company

GFN global financial network

IF investment fund

IFC international financial centre

IFSC International Financial Services Centre

NAV net asset value

OJ offshore jurisdiction

UCITS Undertakings for the Collective Investment in Transferable Securities
